# The effect of friction stir processing on mechanical, wear and corrosion characteristics of Cu-AlN-BN surface composite

**DOI:** 10.1016/j.heliyon.2024.e30173

**Published:** 2024-04-26

**Authors:** Titus Thankachan, K. Soorya Prakash, V. Kavimani, Wenbin Zhou

**Affiliations:** a Department of Mechanical Engineering, Karpagam college of Engineering, Coimbatore, 641 032, India; b Department of Mechanical Engineering, Anna University Regional campus, Coimbatore, 641 046, India; c Department of Mechanical Engineering, Karpagam Academy of Higher Education, Coimbatore, 641 024, India; d Centre for Material Science, Karpagam Academy of Higher Education, Coimbatore, 641 024, India; e School of Science and Engineering, University of Dundee, Dundee, DD1 4HN, UK

**Keywords:** Friction stir processing, Copper, Mechanical strength, Micro hardness, Corrosion behaviour

## Abstract

This research investigates the impact of hybrid particles dispersed onto the surface of a copper matrix using Friction Stir Processing (FSP) on its microstructural, mechanical, and corrosion behavior. The hybrid particles under study consist of equal fractions of Aluminium Nitride (AlN) and Boron Nitride (BN). Microstructural characterization confirms breakdown of grain size due to dynamic recrystallization and presence of particles, along with their effective bonding to copper matrix. Attained results indicated a significant enhancement in hardness, with an increase of up to 3.9 % upon the introduction of particles onto the surface. Moreover, the tensile properties exhibit noticeable improvements in terms of ultimate tensile strength (6.39 %) and yield strength (6.12 %), albeit at the expense of reduced ductility in the copper matrix. Furthermore, the wear rate (decreases up to 22 %) and corrosion rate of the developed composites demonstrate a decreasing trend with the introduction of particles. This improvement can be attributed to the reduction in grain size during the FSP process and the formation of a nitride passive layer facilitated by the reinforced hybrid particles, thereby effectively inhibiting the corrosion rate.

## 1 Introduction

Copper, considered to be an important metal frequently used in diverse industrial applications exhibits an enhanced electrical and thermal conductivity, formability, workability with superior resistance against atmospheric corrosion [1,2]. Even though widely used its application is restricted to a certain limit towing to its low mechanical strength, thermal stability, hardness and wear resistance [[3], [4], [5]]. So as to overcome the above said limitations, researchers prompt to reinforce certain ceramic particles which tend to increase its mechanical properties that include hardness and wear resistance to a great extent [[6], [7], [8]]. These properties equip copper metal matrix as a well-established material in various applications that include thermal, electronic, marine, water utilities etc. [9,10]. Studies proved to increase certain properties of copper with introduction of ceramic particles but dispersion of these particles likely reduces the bulk properties of the developed composite material [11]. To increase the hardness and wear resistance of copper, both being a surface dependent property, modification of surface microstructure or composition may be considered. It has been found that addition of ceramic particles onto surface of copper matrix can improve its the hardness and wear resistance without sacrificing its bulk properties [12,13]. Surface modifications can be carried out through many routes in which laser beam melting, plasma arc melting, thermal spraying etc have been extensively used by researchers. These processes usually operate at high temperatures, leading to unavoidable reactions between the ceramic reinforcement particles and matrix material, thus the expected properties maybe reduced due to the occurrence of certain detrimental phases and intermetallic formations [14,15]. To avoid the above mentioned formations and complications a process that employs anywhere below the melting point of the matrix material has to be considered. Friction Stir Processing (FSP), a novel semi-solid surface modification technique developed based on the methodology of Friction Stir Welding (FSW), is attaining consideration by researchers for microstructure modifications and development of surface composites [16,17]. FSP works under the principle of plastically deforming the base material as a function of the frictional heat generated by the rotating FSP tool, and the continuous stirring action of the same leads to rigorous breaking down of grains with mixing of reinforcement particles throughout the surface.

Researchers have effectively used the forms of carbides, oxides and borides for surface modification of copper matrix through FSP process and the results have promised an optimistic approach tempting to explore the advanced forms of ceramic particles. Nitride based ceramic particles guarantee high hardness, corrosion resistance and tribological property enhancements, promoting them as a candidate material for protective metal coatings [18]. Aluminium Nitride (AlN), an advanced form of ceramic particles, exhibits a unique combination of properties assuring its wide future application in electronic and semiconductor industries. AlN offers high thermal conductivity, electrical break down strength, thermal shock resistance, low specific gravity, enhanced mechanical and corrosion resistance properties [[19], [20], [21]]**.** These properties have converged it as a fitting ceramic particle for protective coating for corrosion resistance [22]. Sager et al. explored the use of AlN particles as reinforcement in metal matrix composites and investigated their effect on corrosion behaviour. The results reveal that the addition of AlN leads to a reduction in grain sizes and enhances the corrosion resistance of the matrix materials [23]. Boron nitride (BN), similar to carbon is an important technological material with a stunning set of properties that include high thermal conductivity, low electrical conductivity, low dielectric constant, excellent thermal stability, and superior resistance to corrosion and oxidation [[24], [25], [26]]. Mathew et al. observed that BN particles effectively suppress electrochemical reactions due to their outstanding barrier performance, stemming from their high insulating characteristics [27].

FSP route leads to severe microstructural modifications on the surface of the base material as a part of plastic deformation and stirring action breaking down of grains sternly. These modifications as stated above can enhance the hardness and wear resistance but can affect an important surface property, corrosion behaviour in a positive or negative aspect. This forces researchers to carry out a detailed study on the electrochemical behaviour of the metal or composite once exposed to FSP surface modification. Literatures regarding studies on corrosive behaviour of FSPed alloys and composites were found to be minimal.

Li et al. investigated the mechanical and corrosive behaviour of FSPed copper. They found that the corrosion rate of FSPed copper was decreased compared to that of as-received copper. Their research revealed that the FSPed copper surface exhibited significantly enhanced wear and corrosion resistance qualities, with improvements of 34.25 % in hardness and 44.76 % in tensile strength. The study attributed these improvements to the rapid formation of a passive layer and the strengthening effect of grain refinement. Furthermore, the FSP route was noted to release residual stress through an annealing process during stirring action, significantly enhancing corrosion resistance [28]. Eivani et al. proved that the corrosion resistance of a material increases with respect to number of FSP passes because corrosion rate is inversely proportional to grain size of the material [29].

Based on the available literature, it is well acknowledged that FSP route increases corrosion resistance of a material to a great extent and at the same time dispersion of an appropriate reinforcement can increase the existing surface properties of copper metal. Thankachan et al. studied the effect of AlN particles FSPed onto the surface of copper matrix and the corrosive resistance was found increasing with respect to an increase in vol% of AlN particles [30]. Similarly the addition of AlN particles onto the surface of copper increases the mechanical strength along with its hardness and tribological resistance. Likewise the addition of BN as reinforcement increases the wear and corrosion resistance of base material. However usage of AlN and BN in hybrid form as the reinforcement in copper surface composite is rarely reported. Hence this research aims to elucidate the influence of hybrid AlN and BN particles on the fundamental and functional characteristics of copper surface composites. Herein hybrid reinforcement with AlN and BN at equal proportion (1:1) is synthesized through a chemical mechanical stirring technique and the same is dispersed over the surface of copper matrix through FSP. The microstructural characterization, mechanical, wear and corrosive behaviour of the material is carried out so as to investigate the effect of FSP and hybrid reinforcement particles on governing the functional properties of copper-based surface composites.

## 2 Materials and methods

### 2.1 Material preparation

**Fig. 1:**
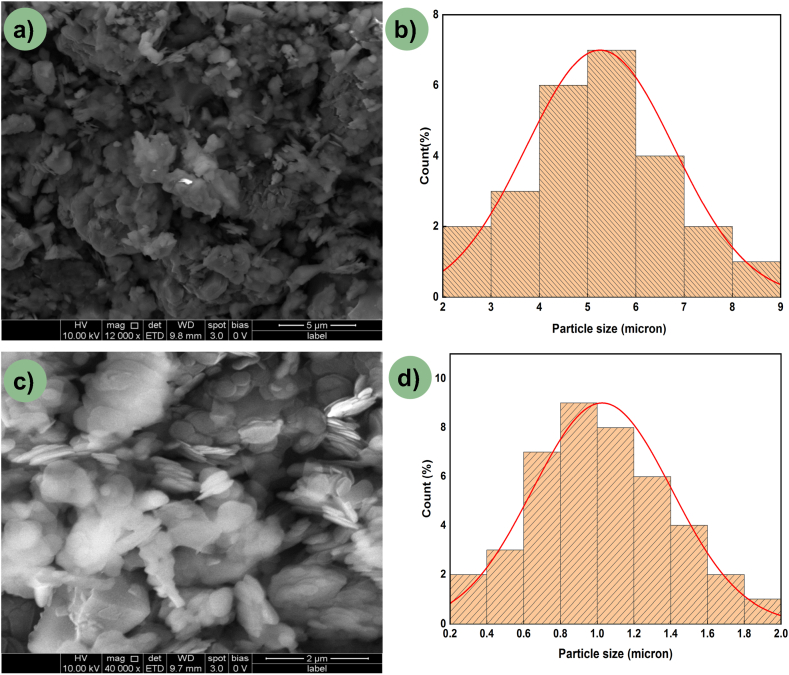
(a, c) SEM micrograph of AlN & BN; (b, d) Particle size histogram of AlN & BN.

**Fig. 2:**
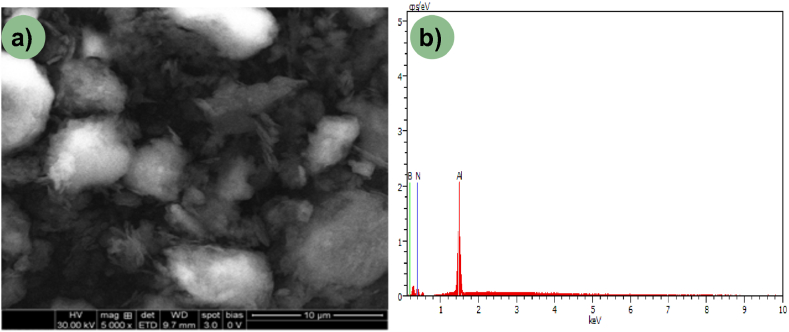
(a–b)EDS spectrum of the hybrid reinforcement.

In this research, an electrolytic copper plate measuring 150 × 50 × 8 mm was served as the base material onto which a hybrid reinforcement was to be dispersed. The reinforcement particles utilized comprised a uniform mixture of AlN and BN particles acquired from Sigma Aldrich. AlN and BN particles, with sizes of approximately 5 and 1 μm respectively (as shown in Fig. 1a–d), were selected for the study. These particles underwent a chemical-based mechanical blending reaction, where both powders were separately mixed with acetone, a non-reactive agent, for an optimized duration and speed using a magnetic stirrer. The solutions containing AlN and BN particles were then blended together in a drop-by-drop manner and thoroughly mixed for a predetermined duration using the magnetic stirrer. The microstructural micrograph, depicted in Fig. 2a, illustrates a thorough mixture of AlN and BN particles. Additionally, the EDS spectrum of the achieved mixture confirms the presence of AlN and BN particles, as shown in Fig. 2b.

### 2.2 Experimental procedure

**Fig. 3:**
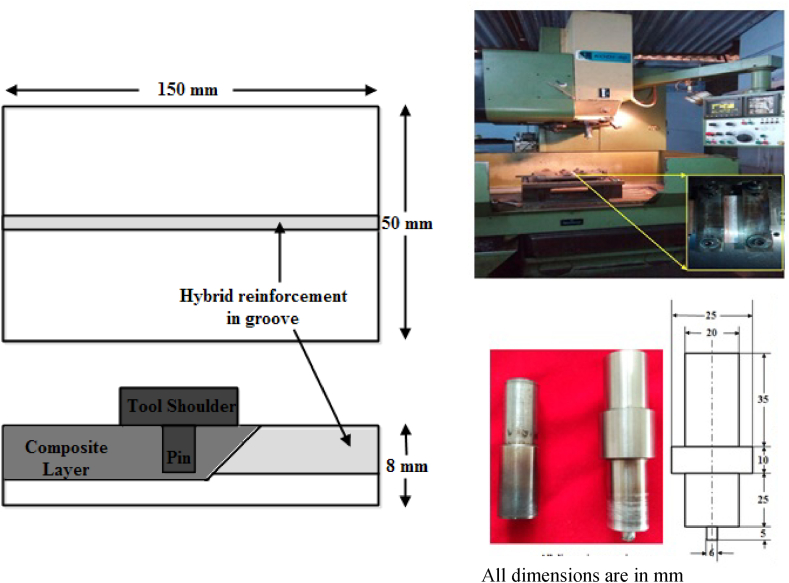
Experimental set-up of the FSP process and the FSP tools.

Fig. 3 illustrates the experimental set-up of the FSP process and the FSP tools. The procedure is such that a groove is cut on the center of copper into which the developed hybrid reinforcement (AlN and BN particles in equal proportion) is compacted. A pinless tool shoulder is first allowed to run above the groove so as to seal it thereby avoiding the sputtering of the particles during FSP. A tool shoulder with pin is then passed on the same position such that the material gets deformed plastically and the reinforcement particles get dispersed into the matrix as an action of stirring. A CNC milling machine with displacement controlling facility was employed to carry out FSP in this research. FSP tool material was opted to be a double tempered H13 steel of hardness 56 HRC, and the tool was of dimensions 20 mm shoulder diameter with a pin diameter of 6 mm and length 5 mm. The FSP processing was carried out at an optimized spindle speed and feed rate of 1000 rpm and 30 mm/min respectively. Various vol% of hybrid reinforcements encompassing AlN and BN particles of equal proportion were dispersed onto the Copper surface through friction stir processing, which will be notified as S1, S2 and S3 for 5, 10 and 15 vol% respectively.

### 2.3 Material characterization

Microstructural characterization of the developed surface composites includes a detailed examination through Leica-DM750 M Optical Microscopy (OM) and Scanning Electron Microscope (SEM). OM provides insight into grain breakdown, while SEM offers clear insight into particle dispersion, bonding, and interface characteristics, as well as the presence of voids if any. Microstructural characterization was carried out after polishing specimens according to metallographic standards using emery sheets of various grades and a velvet disc polisher. Specimens underwent polishing as per the stated procedure, followed by etching with an etchant prepared from an optimized composition of H_2_O_2_, Fe_3_Cl, and distilled water. The etchant was allowed to expose the specimen surface for a specific duration, after which microstructural characterization was conducted. Density evaluation based on Archimedes' principle was performed for the developed copper surface composite by weighing the specimens in air and water, thereby estimating the density values. Micro hardness of the FSPed surface composite was evaluated according to ASTM E384 standards using Matsuzawa-MMTX7 Vickers hardness tester at a load of 50 gm for 15 s at 5 points on the stir zone, with the average considered as the effective hardness of the composites. Tensile specimens were cut from the center of the stir zone to the specified depth according to ASTM E8 standards and tested using an Instron tensile machine at an ambient temperature condition with a strain rate of 10^−5^ s^−1^. Wear testing was conducted according to ASTM standard G99-04 using a pin-on-disc apparatus. Samples were subjected to a sliding wear test against a hardened chromium steel disc with an applied load of 30 N and a rotational velocity of 1 m/s, covering a distance of 1500 m. Wear rate evaluation for surface composite specimens was based on the weight loss of the samples before and after testing. All experimental trials in this research were conducted three times, and the average values were used for result conformity. Electrochemical investigations were carried out on the developed surface composite after polishing to 1200 grit emery sheets. Corrosion behaviour of the composite was investigated under both saline (3.5 % NaCl) and acidic (0.1 M HCl) environments using an AMETEK made VERSASTAT3-400 electrochemical workstation. The electrochemical cell comprised a platinum counter electrode, calomel reference electrode, and the surface composite specimens as the working electrode. The volume of electrolyte was kept constant, and corrosion tests for all three considered specimens were conducted with a constant area of 1 cm^2^ exposed to the electrolyte. Potentiodynamic polarization was performed between 0 V and −2.5 V at a scan rate of 0.01 V/s.

## 3 Results and discussion

### 3.1 Microstructural characterization

**Fig. 4:**
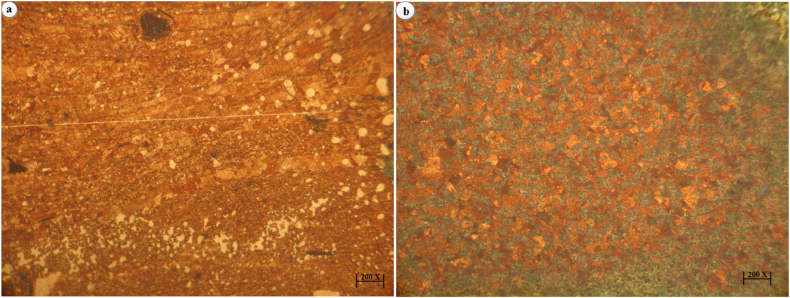
Optical microscope images of (a) 5 vol% reinforcement (b) 15 vol% reinforcement.

The optical microscope images of the developed surface composites dispersed with 5 and 15 vol% of hybrid reinforcements (which include AlN and BN in equal proportions) are shown in Fig. 4a–b. It confirms a severe breaking down of grain size and the same can be explained as an effect of the rigorous stirring action offered by the tool during FSP route. Shoulder of FSP tool creates a friction between work pieces during FSP route increasing the temperature and plastically deforming the material, stirring action offered by pin at this high temperature breaks down the grain size to smaller size. Dynamic recrystallization of matrix happens during FSP route thus reducing the grain size; apart from this the dispersion of hybrid particles also inhibits the grain growth thereby reducing grain size. Difference in particle size along with its smaller radii also leads to reduction in grain size and the same mechanism is termed as Zener–Hollomon mechanism.

**Fig. 5:**
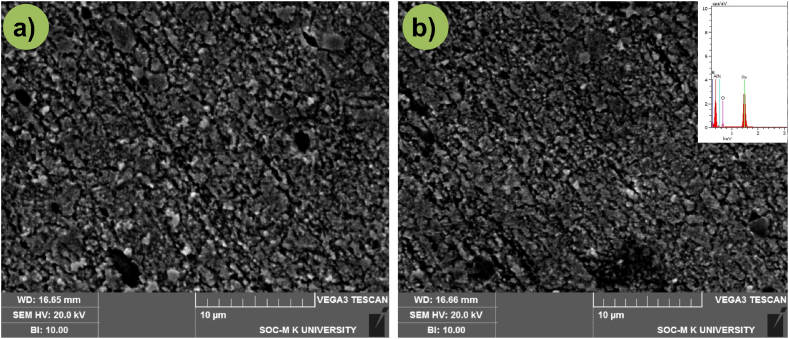
SEM micrographs of (a) 5 vol% reinforcement (b) 15 vol% reinforcement.

In addition to optical microscope images, SEM characterization of the same was carried over so as to investigate the particle dispersion and its bonding between matrix materials, as shown in Fig. 5a-b. It proves that hybrid reinforcement particles have been well dispersed into copper matrix during FSP route offering good bonding with the matrix material. Also, it can be seen that the particles present on the matrix have a clear interface demonstrating the bonding created between particles and matrix material to be clean and without any redundant intermetallics. Irregularities in the size of particles can be observed indicating the breaking down of particles during FSP route.

### 3.2 Density and hardness evaluation

**Fig. 6:**
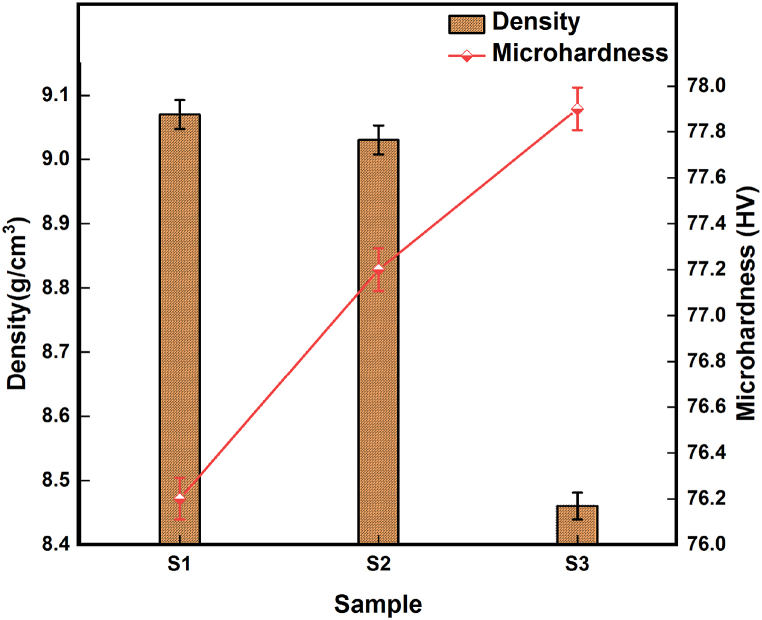
Density and hardness for developed samples.

Density evaluation through Archimedes principle is shown in Fig. 6, it indicates that the increase of the fraction of hybrid reinforcement particles onto the surface of copper reduces the overall density of the developed composites. Compared with density of copper (8.96 g/cm^3^), developed surface composites (S1, S2) exhibit a slightly higher density and this effect may be endorsed as an effect of grain refinement and reduction of the porosity of the developed composites during FSP procedure. Reduction in density with respect to vol% increment is due to the presence of low density AlN and BN particles that gets dispersed onto the surface. Hardness of developed copper surface composites exhibits an increasing tendency with respect to particle addition, as the particles get dispersed onto the surface carrying away the load and thereby reducing the tendency of the material to deform. FSP leads to recrystallization of material which reduces the grain size, and this grain refinement increases the hardness of material as per Hall-Petch correlation. Yet again the work hardening effect of copper and quench hardening effect of surface composite as a function of thermal property misfit between matrix and reinforcement particles also attribute to increase in the hardness value [23,31].

### 3.3 Tensile properties analysis

**Fig. 7:**
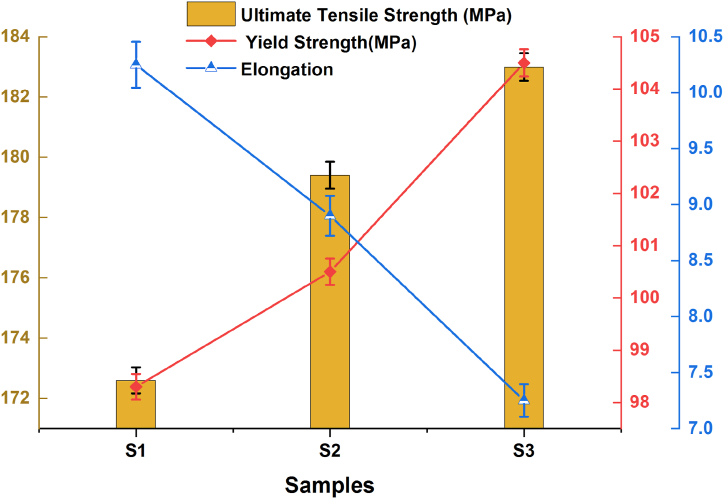
Tensile properties of the developed composite samples.

The tensile properties, including ultimate tensile strength, yield strength, and percent elongation, of the developed surface composites with different volume percentages of hybrid particles are depicted in Fig. 7. The ultimate tensile strength of developed surface composites shows an increasing trend with the introduction of hybrid reinforcement particles into the copper matrix, as illustrated in Fig. 7. This rise in tensile strength can be attributed to the reduction in grain size induced by the FSP process. During FSP, the stirring action induces significant plastic deformation and localized heating, leading to dynamic recrystallization and grain refinement in the matrix material. Finer grains typically yield improved mechanical properties, such as higher strength. Additionally, the incorporation of reinforcement particles further refines the grain structure, augmenting the overall strength of the composite. The hybrid reinforcement acts as load-bearing elements within the composite material. When subjected to external forces, these particles facilitate a more uniform distribution of the load throughout the matrix. With an increasing volume fraction of reinforcement, more particles are available to support the applied load, resulting in enhanced strength. Furthermore, the presence of reinforcement particles impedes the movement of dislocations within the matrix, serving as obstacles that dislocations must overcome. This pinning effect becomes more pronounced with higher volume fractions of reinforcement, contributing to increased strength. AlN and BN particles are typically less ductile than the copper matrix. As the volume fraction of these particles increases, their presence within the composite restricts the ability of the matrix material to undergo plastic deformation. This reduction in ductility occurs because the reinforcement particles resist deformation and hinder the movement of dislocations within the matrix, which is essential for ductile behaviour [21].

### 3.4 Wear behaviour of developed composite

**Fig. 8:**
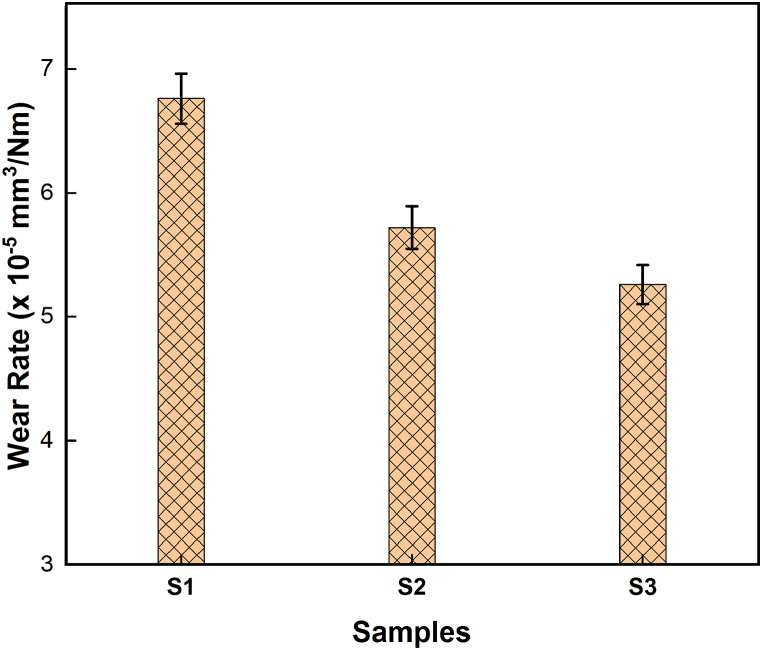
Effect of reinforcement addition on wear rate.

The wear behaviour of the developed composite is illustrated in Fig. 8, revealing that the addition of hybrid reinforcement enhances the wear resistance of the surface composite. Herein, the hybrid reinforcement particles are notably harder than the copper matrix. As the volume fraction of these rigid reinforcement particles increases, they function as resistant elements within the composite. When subjected to wear conditions, these particles offer heightened resistance to wear, thereby reducing the rate of material loss. FSP induces severe plastic deformation and localized heating in copper matrix, resulting in dynamic recrystallization and grain refinement. Finer grain sizes are associated with increased hardness, which can enhance wear resistance. This observation is supported by Archard's law, which stipulates that an increase in hardness leads to a decrease in wear loss. Similarly, BN particles exhibit self-lubricating properties due to their layered structure, which diminishes friction and wear between sliding surfaces. This lubricating effect contributes to an overall reduction in wear rate by minimizing surface contact and frictional forces [32,33].

### 3.5 Corrosion behaviour

The mechanism of cathodic and anodic dissolution of copper material in chloride conditions is through oxygen reduction and conversion of metallic copper to cuprous ion respectively. Cathodic chemical reactions occur as given in Eq. (1):(1)

Copper dissolution through anodic reaction occurs as a chain of reaction in which metallic copper gets oxidised to form cuprous iron, which reacts with chloride ion from electrolyte solution to form CuCl. Resultant CuCl gets transformed to cuprous chloride with further addition of chloride ions and this formed cuprous chloride complex results in the dissolution of copper. Dissolution of copper through anodic reactions is depicted in Eqs. (2)–(4):(2)(3)(4)

**Fig. 9:**
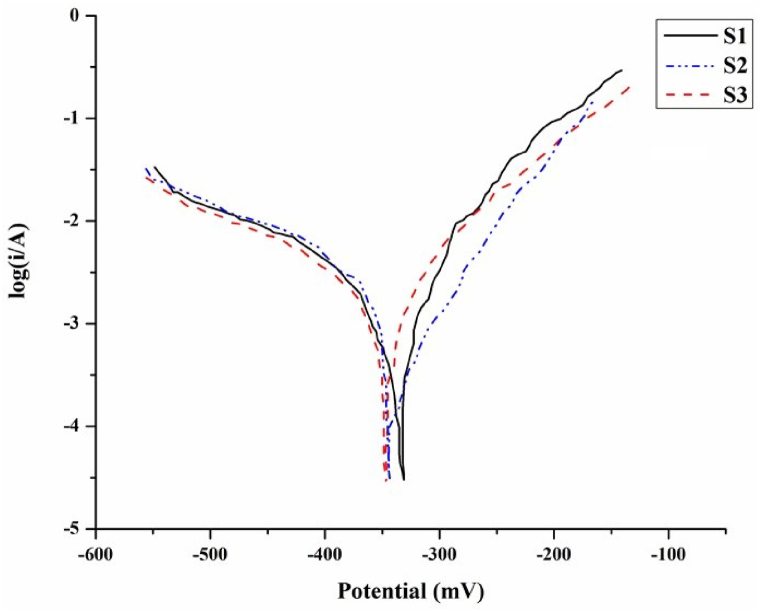
Tafel plot of surface composites at 3.5 wt% NaCl solution.

**Fig. 10:**
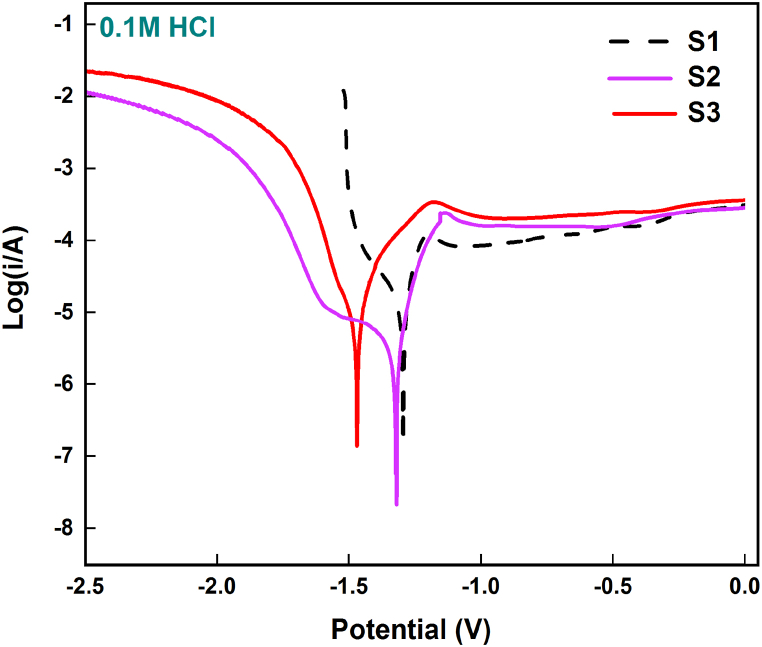
Tafel plots of surface composites at 0.1 M HCl solution.

Polarization curves or Tafel curves are widely employed to predict the capability of a metallic specimen to endure against corrosive environments. The polarization curves for the developed copper surface composites with varying vol. % of hybrid reinforcement particles in 3.5 NaCl electrolytic medium are shown in Fig. 9. Tafel plots of developed composites showcase a slight shifty of curve towards the positive side confirming the anodic reaction. Fig. 10 shows the Tafel plots of surface composites at 0.1 M HCl solution. Electrochemical parameters including the corrosion current (*i*_corr_), and potential (*E*_corr_) attained from extrapolating the Tafel curves of the developed set of surface composite specimens at saline and acidic conditions are provided in Table 1, Table 2, respectively. At saline condition the corrosion potential and current for specimen S1 dispersed with 5 vol % of hybrid reinforcement particles are found to be −342.30 mV and 76.397 μA, respectively, while the corrosion potential for S2 and S3 is −346.31 mV and −349.06 mV respectively. Corrosion current, *i*_corr_ value is evaluated to be 27.135 μA for S2 and 9.812 μA for S3. Likewise at acidic condition S2 displays with an *E*_corr_ of −1.327 V, an *i*_corr_ of 13.5 μA which is slightly in cathodic region when compared with S1 (Fig. 10). S1 exhibits a corrosion potential of −1.295 V and a corrosion current of 37.5 μA, this suggests the potential movement towards anodic region that tends to metal dissolution, as a result the S1 specimen has a higher corrosion rate. S3, on the other hand, shows the most negative corrosion potential at −1.469 V indicating the cathodic region or passive layer formation. A decrease in *i*_corr_ value can be observed with increase in the introduction of the reinforcement particles. Polarization resistance derives the amount of material dissolved during the corrosion procedure and it can be confirmed based on Table 1, Table 2 that copper metal dispersed with 15 vol % of hybrid composites (S3) dissolves the minimal during corrosion testing. This reduction in material dissolution can be concluded as the result of an effective combination of reinforcement particle dispersion along with FSP possessions on material surface.

**Table 1:** Tafel plot results of composite at saline condition.

Samples	*E*_corr_ (mV)	*i*_corr_ (μA)	CR (mmpy)
S1	−342.30	76.397	1.7769
S2	−346.31	27.135	0.6311
S3	−349.06	9.812	0.4120

**Table 2:** Extrapolated values of acidic condition.

Samples	*E*_corr_ (V)	*i*_corr_ (μA)	CR (mmpy)
S1	−1.295	37.5	0.2732
S2	−1.327	13.5	0.1574
S3	−1.469	4.7	0.0546

The corrosion tendency of a material in thermodynamics basis is reflected by the value of *E*_corr_ and *i*_corr_, which is proportional to the corrosion rate of the material. Thus based on the above statement it can be stated that corrosion rate of the developed composite is found decreasing with increasing in vol% of hybrid particle dispersion. To calculate the corrosion rate (CR) of the developed surface composites, the below provided Equation (5) is employed:

(5) $$\text{CR}=\frac{Ki_{corr\;}\text{EW}}{d}$$

Where K denotes corrosion rate constant (milli-inch per year), EW is the equivalent weight of copper and d is its density. Calculated results for corrosion rate value of the copper-based surface composites are shown in Table 1, Table 2, which prove the corrosion rate is reduced with increase in vol. % of reinforcement particles. Increase of corrosion resistance of the developed composites can be attributed to many metallurgical and processing parameters while impending the FSP route. In this research, AlN and BN particles at equal proportion are mixed together and dispersed onto the surface of copper matrix, henceforth along with the corrosion mechanism of copper, kinetics and mechanisms behind the corrosion behaviour of AlN and BN particle ought to also be considered. Yet again, in addition to this, FSP route breaks down the grain size of the matrix material and therefore the corrosion activities of the resultant copper matrix might also change.

Corrosion resistance of the developed composites as a function of AlN particles dispersed onto the surface can be stated such that the presence of nitrides could form a thin oxide passive layer on the surface of the specimens once the particles come in contact with NaCl and HCl electrolyte solution. The presence of higher aluminium content in AlN particles forms a dense and continuous oxide layers composed of alumina as the major compound during the corrosion procedure, thereby inhibiting the degrading of the material during corrosion process [34]. In addition to this AlN particles dispersion leads to reduction in electro migration, thereby reducing the corrosion rate of the developed copper surface composite. BN particles also slow down the corrosion rate owing to their insulating nature which prohibits the electron transport [35].

Studies on the corrosion behaviour of surface modified metals have confirmed that the corrosion rate is directly related to the amount of particles dissolved and the particle distribution among the matrix [36]. FSP route is well renowned in dispersing the particles onto the surface and breaking down the second phase particles. FSP route reduces the porosity of the developed composites and high dislocation density and angle grain boundaries are generated during processing, thereby enhancing the corrosion resistance of the developed set of composites. With increase in the vol. % dispersion of hybrid particles, the grain structure gets refined as a result of dynamic recrystallization, thereby increasing the grain boundary region. This increase in the grain boundary region inhibits the corrosion rate of the developed set of composites.

**Fig. 11:**
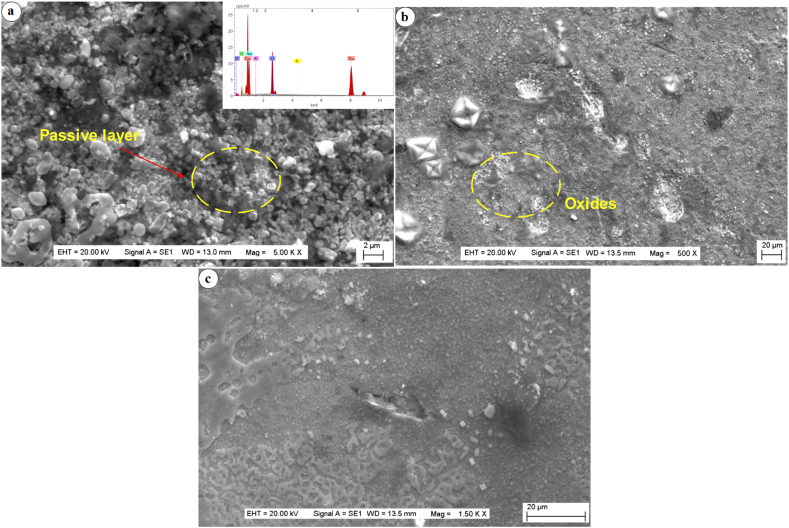
Corroded surface of composite at saline condition (a) 5 vol% reinforcement (b) 10 vol% reinforcement (c) 15 vol% reinforcement.

The examination of the corroded surfaces depicted in Fig. 11a-c offers intriguing insights into the corrosion behaviour of the developed surface composite samples. Notably, a clear correlation emerges: as the dispersion of particles increases, there is a discernible reduction in the extent of corroded areas. Moreover, a striking observation is the minimal incidence of crevice corrosion in the developed surface composites as the dispersion percentage of reinforcements rises. This observation is underscored by the micrographs, particularly evident in micrographs 11(b) and 11(c), where the presence of negligible pits contrasts sharply with the more pronounced corrosion in micrograph 11(a). The mechanism underlying this reduction in corrosion rate is multifaceted. It can be primarily attributed to the presence of AlN and BN particles, which impart a protective/passive nitride layer over the surface, effectively hindering corrosion propagation [37]. Additionally, the increased concentration of AlN and BN particles within the copper matrix contributes to grain size refinement, thereby further mitigating corrosion rates. The role of the Friction Stir Processing (FSP) technique in enhancing microstructure refinement is noteworthy. By breaking down grain sizes and facilitating uniform dispersion of particles throughout the stir zone, FSP minimizes particle agglomeration and precipitation, thus reinforcing the corrosion-resistant properties of the composite. Further confirmation of the composition and corrosion mechanisms is provided by Energy Dispersive Spectroscopy (EDS) analysis of the corroded surfaces. The presence of copper, alongside dispersed aluminium, boron, and nitrogen elements, is indicative of the incorporation of AlN and BN particles within the composite matrix. Moreover, the detection of oxygen in the EDS results corroborates the formation of oxide layers, underscoring the complex interplay of corrosion reactions occurring during the corrosion process.

## 4 Conclusions

This study utilized friction stir processing (FSP) to effectively disperse a hybrid reinforcement comprising AlN and BN particles onto the copper surface. Through a meticulous investigation of the properties of the resultant copper surface composites with varying volume percentages of reinforcement particles, several significant conclusions were drawn. Firstly, the developed surface composites exhibited a finer grain size, attributed to the dispersion of particles onto the copper matrix, which in turn fostered improved bonding and clear interfaces between the particles and the matrix. This structural refinement was accompanied by a notable increase in hardness values as the dispersion of hybrid particles onto the copper matrix increased. This enhancement in hardness was primarily attributed to the efficient distribution of particles and the occurrence of dynamic recrystallization processes, with the lower density of AlN and BN particles further contributing to a reduction in the overall density of the developed surface composite material. Moreover, the mechanical properties, including ultimate tensile strength and yield strength, displayed a positive correlation with an increase in particle dispersion, driven by the reduction in grain size. However, it was observed that ductility tended to decrease with the addition of reinforcement. Additionally, the incorporation of reinforcement particles led to a significant decrease in the wear rate of the composites. Notably, potentiodynamic polarization curves for the developed surface composites revealed a notable improvement in corrosion resistance with an increase in the volume percentage of hybrid reinforcement particles. This enhancement was attributed to grain refinement induced by FSP and the formation of a protective nitride layer over the composite surface.

## Data availability statement

Data will be made available on request.

## CRediT authorship contribution statement

**Titus Thankachan:** Writing – original draft, Software, Investigation, Formal analysis, Data curation, Conceptualization. **K. Soorya Prakash:** Validation, Software, Resources, Investigation, Data curation. **V. Kavimani:** Writing – review & editing, Validation, Software, Investigation, Data curation. **Wenbin Zhou:** Writing – review & editing, Visualization, Resources, Methodology, Investigation, Conceptualization.

## Declaration of competing interest

The author W.Z. is an Associate Editor for Heliyon and was not involved in the editorial review or the decision to publish this article.
